# Factors influencing the long-term sustainment of quality improvements made in addiction treatment facilities: a qualitative study

**DOI:** 10.1186/s13722-017-0093-x

**Published:** 2017-11-01

**Authors:** Scott P. Stumbo, James H. Ford, Carla A. Green

**Affiliations:** 1 Kaiser Permanente Northwest, Center for Health Research, 3800 N. Interstate Avenue, Portland, OR 97227-1110 USA; 2 Center for Health Systems Research and Analysis, University of Wisconsin – Madison, 610 Walnut Street, Madison, WI 53726 USA

**Keywords:** Quality improvement, Long-term sustainment, Addiction treatment, Organizational behavior, NIATx200, Qualitative methods

## Abstract

**Background:**

A greater understanding of the factors that influence long-term sustainment of quality improvement (QI) initiatives is needed to promote organizational ability to sustain QI practices over time, help improve future interventions, and increase the value of QI investments.

**Methods:**

We approached 83 of 201 executive sponsors or change leaders at addiction treatment organizations that participated in the 2007–2009 NIATx200 QI intervention. We completed semi-structured interviews with 33 individuals between November 2015 and April 2016. NIATx200 goals were to decrease wait time, increase admissions and improve retention in treatment. Interviews sought to understand factors that either facilitated or impeded long-term sustainment of organizational QI practices made during the intervention. We used thematic analysis to organize the data and group patterns of responses. We assessed available quantitative outcome data and intervention engagement data to corroborate qualitative results.

**Results:**

We used narrative analysis to group four important themes related to long-term sustainment of QI practices: (1) finding alignment between business- and client-centered practices; (2) staff engagement early in QI process added legitimacy which facilitated sustainment; (3) commitment to integrating data into monitoring practices and the identification of a data champion; and (4) adequate organizational human resources devoted to sustainment. We found four corollary factors among agencies which did not sustain practices: (1) lack of evidence of impact on business practices led to discontinuation; (2) disengaged staff and lack of organizational capacity during implementation period led to lack of sustainment; (3) no data integration into overall business practices and no identified data champion; and (4) high staff turnover. In addition, we found that many agencies’ current use of NIATx methods and tools suggested a legacy effect that might improve quality elsewhere, even absent overall sustainment of original study outcome goals. Available quantitative data on wait-time reduction demonstrated general concordance between agency perceptions of, and evidence for, sustainment 2 years following the end of the intervention. Additional quantitative data suggested that greater engagement during the intervention period showed some association with sustainment.

**Conclusions:**

Factors identified in QI frameworks as important for short-term sustainment—organizational capacity (e.g. staffing and leadership) and intervention characteristics (e.g. flexibility and fit)—are also important to long-term sustainment.

**Electronic supplementary material:**

The online version of this article (doi:10.1186/s13722-017-0093-x) contains supplementary material, which is available to authorized users.

## Background

Funders and stakeholders are increasingly asking for evidence that public health investments have meaningful effects that are sustained over time. Recent research has provided some evidence of the impact and sustainment of health interventions [1–3], and leaders in the field have proposed an agenda for additional public health sustainability research [4]. To date, interventions to improve organizational performance and increase capacity to deliver medical and behavioral health interventions often show no results or mixed results, or show short- but not long-term improvements [2, 5, 6].

Broadly defined, sustainment is the maintenance of program components or outcomes once an initial intervention is completed, or funding is withdrawn [1]. In recent years, researchers have improved methods for assessing sustainability and in the process have described factors that increase the likelihood of sustainability or the capacity to sustain improvements [7, 8]. Three factors important to sustainment have been described across numerous studies: agency characteristics, intervention characteristics, and the external environment. Agency characteristics include staff stability [9], leadership [10], the presence of intervention champions [11], and the capacity to routinize innovations and processes [12]. Important intervention characteristics include the value of innovations to the agency [13], and the flexibility to adapt intervention components to fit within an agency [14]. Finally, external factors include funding availability [13] and institutional climate [12]. How these factors coalesce into sustainment, and in which circumstances each component is a necessary precondition for sustainment, is a matter of debate [4, 7, 14, 15]. Further, many studies of sustainment have focused on a relatively short period of time directly following intervention completion. However, some studies have utilized interviews or administrative data to examine sustainability one to 3 years post-intervention [16–18] or even over a longer-term [19–21].

NIATx200 (formerly the Network for the Improvement of Addiction Treatment) was designed to increase the organizational capacity of addiction treatment centers to reduce waitlists for services, increase enrollment, and improve retention of clients engaged in services. The intervention included four separate intervention arms: (1) “coaching” which included a process improvement expert working directly with each agency; (2) “learning sessions” which brought participants together in twice yearly conferences involving process change experts; (3) “interest circle calls” which used monthly conference calls to discuss process improvement activities; and (4) a “combination” arm which included access to all three intervention activities. 201 agencies that admitted > 60 clients in the prior year and received public funding were randomized [22]; 82% of agencies were privately owned and 83% were located in urban areas [23]. All participating agencies had access to the same web-based toolkit, which contained specific instructions on how to conduct a walk-through of the agency to identify improvement opportunities [24], how to use Plan-Do-Study-Act (PDSA) cycles to identify and enact changes, and a list of promising practices specific to each outcome. Agencies in all intervention arms were free to try whichever promising practices were both practical for their agency or they believed would have the most impact. The intervention arms varied on the type and amount of support provided to each agency to adapt and implement promising practices and other intervention tools. Final intervention results showed variability in associations between intervention type and the three main outcomes: (a) coaching, learning session and combination arms were associated with reduced wait time; (b) coaching and combination arms were associated with improvement in admissions; (c) interest circle calls had no association with any outcome; and (d) no intervention type improved patient retention [25]. An exploratory analysis (unpublished) did not identify any significant relationships between the level of agency participation in their assigned NIATx200 intervention arm and improvements in outcomes.

The goal of the current study was to assess from participating agencies which, if any, organizational practices or outcomes were sustained 6–7 years following the completion of the NIATx200 intervention. We wanted to learn what internal organizational factors were important to sustaining practices implemented during the intervention, and what internal factors served as barriers to sustainment. The interviews were also meant to provide context for, and enhance interpretation of, quantitative sustainment outcome and methods data being assessed as part of the larger follow-up project [26]. The study team was interested in external barriers to sustainment encountered by agencies in the post-intervention period, but the variability across state and within-state (e.g. county) policies and payment methods coupled with our relatively small sample size made it difficult to generalize those barriers. In this paper we describe agency and intervention characteristics associated with long-term sustainment of QI practices.

## Methods

### Recruitment

We recruited individuals whose agency participated in the NIATx200 intervention. In most cases this was the executive sponsor of the intervention within the agency or the “change leader” (who was the champion during the intervention). Interviews were conducted between November 2015 and April 2016, approximately 6–7 years after the intervention program ended in 2009.

We identified 83 potential interviewees using a convenience sample with a focus on ensuring representation from each of the four original intervention arms. Our initial goal was to recruit individuals from only 3 states (those with the most complete administrative data available for quantitative verification) but we were unable to meet our recruitment goals and opened up interviews to agencies from the two additional states that participated in the NIATx intervention. We completed interviews with 33 individuals (approximately a 40% response rate); we stopped recruiting when we felt we had reached sufficient saturation and no new codes or themes emerged. See Additional File 1 for a recruitment diagram. Reasons for refusing to participate included: (a) lack of interest or time, (b) not remembering enough about the intervention, or (c) no staff member left at organization who participated in NIATx. Though these last two reasons were not specifically disqualifying, we were unable to convince some potential participants that their insights would still be valuable. Interviews were conducted by phone and typically lasted 45 min. Participants received a $10 gift card to a national coffee chain as a token of appreciation. The University of Wisconsin Institutional Review Board approved and monitored the study.

### Interview guide

We developed the semi-structured interview guide to first ground interviewees in the original intervention by having them describe their role during the intervention and elaborate on their recollections of what transpired during the intervention. Second, we reviewed a list of promising practices with participants who could describe their current use of those practices and also their use during the intervention period. The list of promising practices was organized by outcome (wait time, retention, admissions) and was developed as part of the NIATx200 study [22]. The promising practices were originally provided to participating agencies during the intervention period. It included practices which agencies could adopt during the participation period to improve each outcome, but were meant merely as suggestions. Agencies were free to use other practices to achieve outcome goals. During the interviews, the list of promising practices were a helpful trigger for placing the individual back in the intervention context. Further questions assessed (1) what changes the organization had maintained at the time of the interview that they implemented as part of NIATx200 participation; (2) what changes were made as part of intervention participation that had not been maintained; (3) attributes of practices that made some more sustainable than others; (4) infrastructure changes made to enhance sustainability; and (5) other barriers or facilitators of sustainment. The interview guide is available from the authors upon request.

### Qualitative analytic approach

All interviews were conducted by phone, recorded using encrypted audio-recorders, and transcribed verbatim. Following a pilot test of the interview guide, and completion of the first five interviews, we began reading transcripts to develop the codebook. Other than the general concept of “sustainment” we had no a priori codes in mind when reviewing transcripts, and did not use conceptual domains from extant literature to develop codes to fit participant narratives into predetermined concepts. This modified grounded theory approach [27, 28] encouraged us to use open coding techniques to establish codes and definitions [29]. Open coding began with reading text and noting the broad concepts expressed. In the process we wrote short memos outlining how we believed those concepts were conveyed in the text [29]. From this we created a list of descriptive codes to be applied to the narratives using Atlas.ti [30]. Descriptive codes were applied to portions of text that offered examples, or contra-examples, of concepts that were relevant to themes of sustainability [31]. We developed definitions for all codes once the final list of descriptive codes was complete. We wrote brief case summaries for all 33 completed interviews. The lead author developed initial codes and was responsible for coding all interviews. We used a consensus process (SS and CG) to further refine codes and to assure that coded text aligned with established code definitions.

The results presented in this paper come from the case summaries and focused queries of coded text on topics that participants reported as important to sustainment. Using thematic analysis [32, 33], and elements of grounded theory [27, 29, 31] and constant comparative methods [27], we re-read all queries, searching for patterns in the narratives. This allowed us to develop sub-themes to help explain elements or processes that participants believed were related to intervention sustainment (or lack of sustainment). The themes presented here were developed by sticking closely to the narrative data we collected. No single threshold was used to determine the themes; salience to interviewees and their belief in themes’ relationship to sustainment was critical. The lead author developed initial themes; CG and JF provided refinement of themes and helped situate findings in the sustainment literature.

### Quantitative data

We used available quantitative data to provide a face validity check on our narrative findings, and to contextualize our interviewees as a subset of all intervention participants. First, as a check on narrative sustainment categorization we used observed wait-time outcome data. “Sustainers” were defined as those with a shorter wait time post-intervention compared to baseline values with a *p* value < .05. We calculated the level of long-term engagement with NIATx by assessing the mean level of survey responses (a post-intervention activity) up to 27 months following the intervention. Higher responses indicated greater continued engagement with NIATx. We also calculated participation level in the original intervention using number of sessions attended (arm-specific), and used those results to compare interviewees with individuals we did not interview, and also to compare sustainers versus non-sustainers in our data. All t-tests were calculated using SPSS v.22 (IBM).

## Results

We completed interviews with 25 women and 8 men representing agencies in five states, including nine individuals whose organizations had received the “coaching” intervention during the main trial, seven from the “learning session” arm, ten from the “interest circle” arm, and seven who were in the “combination” arm.

Written case summaries allowed us to group the 33 interviews into two overall categories: agencies which reported sustaining improvements (*n* = 13) and those which reported low/no sustainment (*n* = 20). Agencies were coded as sustainers by using their stated beliefs that they had sustained practices initiated during the intervention period, and that those sustained practices were associated with sustained (or improved) outcomes over time. Sustainment of practices and/or outcomes could have come in any of the three outcome goals (wait-time, retention, admissions) but did not have to be uniform across all three. We corroborated these findings by using data measuring reduction in wait-time for 20 agencies (those with sufficient data approximately 3 years following completion of the wait-time reduction portion of the intervention) to assess congruence with the narrative data. We found that 16 of 20 agencies reported narrative data which matched the measured wait-time data: there was agreement in six cases where both the narrative and observed data pointed to overall wait-time reduction sustainment and agreement in 10 cases where both the narrative and observed data suggested a lack of sustainment. We found four cases where the narrative data suggested sustainment that was not corroborated by the numerical data. Our final results, therefore, include nine agencies which reported sustainment (6 agencies where the reported and observed data were in agreement, and 3 agencies with reported sustainment). See Table 1 for additional information.

**Table 1:** Agreement between narrative description of sustainment and data measuring post-intervention wait-list reduction by intervention arm

	Observed wait list data (n = 20)		Observed wait list data not available (n = 13)
	Sustained	Not sustained	
*Narrative*			
Report sustained	3 combination 1 coaching 1 interest circle 1 learning session	2 interest circle 1 combination 1 coaching	2 coaching 1 learning session
Report not sustained		4 interest circle 2 combination 2 coaching 2 learning session	3 coaching 3 learning session 3 interest circle 1 combination

The post-intervention measurement period was more than 2 years following the cessation of all intervention activities, and more than 3 years after the completion of the intervention segment where wait-time reduction was the primary focus. For the observed data, “sustainers” were defined as those with a shorter wait time post-intervention compared to baseline values with a *p* value < .05. Thirteen agencies we interviewed did not have sufficient data available from that time period to categorize. Later time periods had even greater amounts of missing data

Our analyses of the narrative data were grouped to describe four overarching factors that influenced the long-term sustainability of programmatic investments made during NIATx: impact on business practices; staff engagement; data integration into monitoring activities; and organizational human resources devoted to sustainment. In order to highlight how each of these themes operates within sustaining and non-sustaining agencies, we present the results by sustainment status to describe how each of the four factors influenced or hindered sustainment.

### Agencies that sustained improvements

In this section we describe the role played by the four factors we identified in agencies which sustained improvement after the intervention had ended (n = 9).

#### Impact on business practices: finding alignment between business-centered and client-centered practices

Agencies that reported sustainment described finding an affinity between NIATx’s client-centered principles and the organization’s business practices, and most agencies reporting sustainment had adopted client-centeredness as a core value. As described by one sustaining agency, NIATx encouraged the agency to think about business practices and client-centeredness together, rather than as mutually exclusive goals:

“I would say that it was jointly client-focused and organization-focused. It sort of allowed us to say it’s okay, as an organization, for us to think about our business, and for us to be focusing on the business case for doing these things as opposed to just doing good in the community…We’re allowed to be a business. And we want to be a successful business…And providing good customer service and meeting clients’ needs and being a good business can be the same” (Agency#1, sustainer).

#### Staff engagement: early staff buy-in added legitimacy; legitimacy helped with sustainment

Successfully engaging staff in intervention and QI processes is often critical for their immediate success. Several sustaining agencies reported that getting staff buy-in, early in the process, was critical to legitimizing the roll-out of the intervention in what could have been seen as simply a leadership or top-down business decision. Without such staff engagement some were doubtful that the intervention principles would have taken hold in the first place or been sustained in the long run. As one participant explained, knowing when to broaden the QI team to add legitimacy was critical:

“I was running up against a lot of resistance from staff and other people. [So] I brought onboard our clinical supervisor…who was a very influential leader…[and] had that ability to bring people onboard with these changes and reduce some of this discomfort…I was banging my head up against a wall for a long time before I finally realized I need other support to kind of bring the buy-in from the staff…I just think that they’re more comfortable working together in that way as opposed to me coming to them and saying, hey, I want to try this because I think it will really help improve services” (Agency #6, sustainer).

Bringing existing, respected staff onto the change team gave the agency a long-term work group in which to discuss “shifting workload around and establishing priorities of services…then once we know [something is] a good practice, we just do it.”

#### Embedding data integration: making the connection with quality improvement

Agencies made use of a “change project form” to implement promising practices by identifying areas for improvement, proposing changes, assigning responsible parties, engaging in PDSA steps, and documenting results of the rapid test cycle. Collecting and monitoring change project data were key components of the NIATx intervention; agencies were encouraged to develop simple tools (pencil/paper, spreadsheets) to monitor the impact of their change projects. Study researchers did not provide feedback to the agencies about their outcome performance during the active intervention period.

In our interviews, agencies that reported sustaining intervention improvements described how deeply embedded the philosophy of this data collection and monitoring became during, and following, the intervention period. Some agencies described an individual who was their data champion, and that this role facilitated sustainment. Several agencies reported that monitoring data closely and consistently allowed them to get ahead of any problems (e.g. an increase in waitlist time), rather than falling behind the problem.

“So in the past, we would see a drop in revenue and say, oh, what’s going on…We really learned, as a result of NIATx, that we need to do this on a very consistent basis. And this has become a lot of my job—I’m looking all the time; I’m pulling data. And when I see that there’s an issue, we will say, okay, where do we think it is? What is the date it’s showing [as] of? And then we take a look at that and say, okay, what do we need to tweak? Or what do we need to improve?…[This is] primarily now what I do…it’s something that we definitely prioritized…probably three quarters of [my] job is now doing something related to [data]” (Agency #18, sustainer).

#### Committing organizational human resources: building on the initial investment

Organizational leadership and commitment of human resources also played a role in sustaining changes. Though the NIATx intervention encouraged agencies to develop a “sustainability plan,” no one we interviewed reported that their agency actually developed such a plan. Absent such a plan, one interviewee described the agency’s initial investment in NIATx and the leadership’s subsequent expectation to see something develop from that investment. This participant describes how the agency established and maintained new procedures related to expanding walk-in hours:

“I think…it was the accountability. I really do…I think it was an investment in [organization] that they [leadership] wanted…You know, they’d kind of given up these resources [to accommodate NIATx]. And they were focusing on it…everybody was invested in it. We spent a long time…making sure that we wanted it to work…And, you know, we prioritized it. I think that was the big piece…” (Agency #8, sustainer).

### Agencies that did not sustain improvements

We found four important factors—many the inverse of positive factors associated with sustainment noted above—common among agencies (n = 24) reporting lack of sustainment.

#### Impact on business practices: lack of evidence of impact on bottom line led to discontinuation

For some agencies the lack of ability to sustain improvements was related to lack of evidence that it continued to have an impact on the bottom line. One agency reported that doing reminder calls worked during the intervention, but that it was too labor-intensive to continue given that it is not a reimbursable activity.

“And we’re operating in the black…just barely…And we do that by being very sparse on our admin and management staff…[And] we were tracking our no-show rate, which was generally under 20%…sometimes down around 12%…even without doing the [reminder calls] anymore. So we weren’t feeling like that’s the most urgent thing we had to deal with” (Agency #14, non-sustainer).

#### Lack of agency capacity or lack of buy-in during implementation period led to lack of sustainment

In the course of implementing the NIATx intervention, managers often asked staff—from frontline administrators to counselors—to take on new tasks in order to improve efficiency. Without a general buy-in on intervention goals, however, these requests were sometimes met with resistance. From our interviewees’ perspectives (all of whom were managers), some felt that staff were resistant to shifting work onto their plates, such as requiring them to make the reminder calls. For example, after saying that doing reminder calls “proved onerous,” one interviewee went on to say: “Well, the front desk didn’t like doing it. They usually didn’t have the time to do it. And counselors resisted doing it” (Agency #13, non-sustainer).

In another agency, the lack of tone-setting to get organizational buy-in came directly from the top. Following randomization to the “interest circle calls,” one agency CEO became uninterested in the intervention: “We were told that we would get the face-to-face [coaching] intervention.” Following that, her agency did not participate much in the program. She described the interest circle calls as “worthless” and of the PDSA cycle she said “We all got that [training], but we didn’t do it.” This lack of leadership commitment to NIATx ensured that the agency subsequently devoted no time or resources to engaging in the intervention (Agency # 25, non-sustainer).

Finally, some agencies simply lacked capacity to expand practices, especially practices that staff assumed would increase their workload. For example, when asked why providing walk-in hours did not work, one interviewee said:

“Generally, it was fear on the part of clinicians that they would become overwhelmed…To increase access, doesn’t that mean that my case load will triple?…Clinicians, by and large, are [saying] I can’t make the time…My caseload is full. I can’t see anybody else” (Agency #13, non-sustainer).

#### Running into data roadblocks

Several agencies that were unable to sustain improvements struggled with continued access to data that would allow them to monitor ongoing activities. For some it was that the data was never quite how they needed it; it required too much “massaging” to make it useful. Some agencies mentioned competing reporting requirements from different funders, which took up a lot of time but still did not leave them with the data they really wanted. When describing what she thought her agency needed to sustain or regain improvements made during the intervention period, one interviewee reported:

“More data. More data on the access center, more data on the call volumes, more data…there’s some things that we could track that we’re not tracking because of priority and limitations to resources. So in a perfect world, I would be able to quadruple the IT Department and have a couple of guys in there who really knew how to write these reports in our electronic health record…And maybe someday we’ll get there…I know that this organization really wants to be data driven and to use that to make decisions…” (Agency#11, non-sustainer).

#### Organizational human resources: high levels of staff turnover

If committing organizational human resources is important to sustainment, then high staff turnover is problematic for sustainment. Turnover at community-based agencies is a problem for many organizations. Staff turnover at all levels can be problematic, but agencies reported that it was particularly difficult to sustain improvements when their data analyst left:

“At the time, we had someone who was our data person who was really good with the files and medical records. And she’s no longer with us…[That’s why] there’s not actual data as much…It’s more monthly, quarterly or annually, which is not rapid change…” (Agency #16, non-sustainer).

Further evidence of the relationship between staff turnover and low reports of sustainment in our data can also be seen in the interviews we conducted with individuals (n = 6) who were not there during NIATx intervention period. These individuals agreed to be interviewed because no one currently employed at their agency participated in NIATx. All six agencies were in the non-sustainment group, suggesting anecdotally that lack of organizational staff continuity is inconsistent with sustainment.

### Sustaining the principles if not the outcomes: the legacy of the NIATx intervention

In addition to coding sustainment of specific practices and outcomes, we coded any mention that an agency might still be adhering to any of the guiding principles of NIATx (e.g. using the PDSA cycle, doing regular walk-throughs to identify areas for improvement, using the change project form to assess new activities). We found that 50% (n = 12) of agencies which we classified as non-sustainers still mentioned one or more philosophical tenets of the NIATx intervention as being used within the agency. One individual, who was not at the agency during the intervention period, said:

“We have quarterly meetings to discuss business strengths and weaknesses and…address areas of concern or risk…[O]ne approach that we frequently used these quarterly meetings to explore was Plan, Do, Study, Act, which is a NIATx thing…So that was sort of a nice framework for us to…identify areas that were not as effective or that we felt needed our attention, and then do brief… periods of determining whether any long-term changes would benefit us” (Agency #17, non-sustainer).

Another interviewee reported:

“What I really liked about NIATx is…that we got that worksheet [change form]…We’ve always had some quality improvement we use [here]. But we didn’t document [it]…it wasn’t so formal…The form itself really helped put structure to it. Otherwise, it was kind of chaotic [for] us. So we use that form even today” (Agency #16, non-sustainer).

Finally, it should be noted that agencies (both sustainers and non-sustainers) did report on external barriers to sustainment. However, due to the relatively limited sample and the within- and between-state variability of policy and payment systems, it would be difficult to characterize coherent themes. For example, there were roughly equal numbers of agencies reporting that the implementation of the Affordable Care Act was either a positive change in environment (in that it expanded the eligible population and thus could increase admissions) or a negative change (in that many states capped Medicaid payments at low rates). Some agencies reported that it was both helpful in increasing admissions and unhelpful in reducing overall revenue.

### Quantitative analyses

We conducted additional analyses using available data to assess the generalizability of our narrative data. First, as a measure of overall engagement in the intervention and whether that differed among individuals we enrolled for interviews, we tested the survey completion rate at each of four survey time periods. Participating individuals within all agencies were surveyed at four time points (baseline and three additional times at 9-month intervals) to assess their use of promising practices within their agency. Individuals who agreed to participate in our interviews were more likely to be from agencies with high survey participation rates at both 18 months (mean survey completion = 7.1 vs. 5.6, *p* = .042) and 27 months (mean survey completion = 6.5 vs. 4.7, *p* = .021) following baseline, suggesting an association between higher levels of continued intervention engagement and willingness to participate in an interview (Table 2).

**Table 2:** Association between agency survey completion and interview participation, n = 194

	Agencies interviewed (n = 33)	Agencies not interviewed (n = 161)	*p*
	Mean (SD)	Mean (SD)	
Baseline	8.2 (3.8)	7.2 (3.5)	.134
+ 9 months	6.3 (3.6)	5.4 (4.1)	.257
+ 18 months	7.1 (3.9)	5.6 (4.0)	.042
+ 27 months	6.5 (4.5)	4.7 (4.0)	.021

Surveys asked agency staff about the use of promising practices and other measures of intervention engagement. Multiple respondents at each agency were encouraged. Means represent the number of agency staff completing surveys at each time period

Next we tested whether individuals who participated in our interviews were more or less likely to be from agencies which were more engaged in their respective intervention arms. NIATx measured the amount of engagement as the total participation activity, by arm, relative to the amount of intervention activities offered. Table 3 column A shows a secular trend toward higher levels of engagement in the intervention comparing current interviewees to non-interviewees, but they did not differ statistically.

**Table 3:** Associations between intervention engagement and interview status, and between intervention engagement and sustainment among interviewees

Intervention arm (maximum number of engagement activities in arm)	A			B		
	Among all agencies, n = 201			Among interviewees, n = 33		
	Agencies interviewed (n = 33)	Agencies not interviewed (n = 168)		Sustained improvements (n = 9)	Did not sustain improvements (n = 24)	
	Mean	Mean	*p*	Mean	Mean	*p*
Learning sessions (max = 3)	2.57	1.96	.163	3.00	2.44	.119
Interest circle (max = 18)	6.20	5.05	.408	9.25	5.85	.136
Coaching (max = 18)	12.50	10.26	.201	14.29	11,20	.101
Combo (max = 39)	22.43	17.15	.084	27.33	18.75	.045

Finally, we tested the association between intervention engagement and sustainment status among agencies we interviewed. For three intervention arms we saw higher arm-level engagement among sustainers compared with non-sustainers, though the results were not significant. Among participants in the combination arm, we found higher levels of intervention engagement among those who were able to sustain improvements (27.33 vs. 18.75, *p* = .045). See Table 3 column B for additional results. This suggests that a high level of engagement in intervention activities among those assigned to the combination arm may be associated with sustainment.

## Discussion

We found variability in sustainment at addiction treatment facilities 7 years after the completion of a quality improvement intervention. Four factors were found to influence long-term sustainment: impact on business practices, staff buy-in and engagement, an organizational commitment to sustainment, and the ability to embed new data processes into an overall organizational QI strategy. These findings on the long-term sustainment of intervention improvements share several common factors with previously published studies on short-term sustainment, and sustainment in other organizational contexts. For example, our findings on the importance of intervention characteristics to sustainment are similar to those described elsewhere [2, 7, 9]. Specifically, our work documents that the intervention’s positive impact on business efficiency, and concomitant improvements to the bottom line, was more common among agencies which sustained practices.

Also similar to findings from short-term sustainment studies, we found that agency capacity played a critical role in long-term sustainment of intervention effects [2, 9–11]. In our study this included the capacity to elicit staff buy-in and engagement early in the intervention [13, 34], and the capacity to sustain staffing to maintain practice changes. Conversely, staff turnover, previously identified as a barrier to implementing an intervention [35] and also as a barrier to sustainment [2, 13], was a factor related to non-sustainment in our findings. We found that no agencies reported documenting all intervention efforts for future staff thus underscoring the importance of staff continuity.

Our finding on the importance of the use of data to monitor and improve activities demonstrates the dynamic interplay between intervention characteristics and agency capacity, and their relationship to sustainment. Data collection to monitor and review intervention-related improvements was a key characteristic of the intervention for all participating agencies. An agency’s capacity to develop and maintain the person who could serve as a data champion was also critical. However, the ability to sustain data monitoring efforts was related not just to the person but to a process. Agencies which embedded data review into their QI teams, or used intervention participation to spur the creation of such a team, were more likely to report sustaining improvements over time. Some agencies found they had an individual with this capacity while other agencies nurtured the development of a person to play this role; agencies who lacked such a person, or who lost their data person, showed a lack of sustainment. The importance of a champion, in this case a data champion, is something that others have noted as critical to the routinization of sustainment [7, 9, 11].

Our quantitative results she light on additional areas of interest to the sustainment field. Intervention arm assignment did not show a strong association with sustainment status in our data; agencies enrolled in all four arms were distributed across the sustainer and non-sustainer groups. NIATx practices were available to all agencies regardless of intervention arm, and some agencies sustained practices (e.g. use of PDSA cycle) despite lack of overall sustainment and independent of the facilitative support offered as part of the intervention arm. These findings suggest that the relationship between intervention strength, intervention engagement, and sustainment is complex. Sustainment may best be seen as a dynamic process on a continuum rather than as all or nothing at a fixed point in time, something others have noted [2]. Agencies which sustained practices, regardless of intervention arm, may share another trait in common: agency capacity. Certainly our results demonstrate the overall importance of agency capacity in getting staff buy-in and maintaining staff, including a data champion. Future research could explore, quantitatively, the relationship between intervention engagement, agency capacity, and long-term sustainment.

Finally, we found that the total “impact” of an intervention is difficult to measure. For example, how should one define and measure an agency’s perception that tools and methods learned during the intervention (e.g. using PDSA or walk-throughs to improve services), are still important to the agency and, in fact, still being used, even absent measureable improvement in the outcomes studied? Agencies, and researchers, wishing to extend intervention investments need to understand the value of continuing any portion of an intervention, and how to measure those latent effects [12]. Such improvements to measuring the full impact of an intervention could focus not just on the diffusion and replication of activities in other settings [4], but on other QI activities within the same organization. Future work could explore identifying unmeasured effects of sustained intervention practices, or measuring sustainment of QI practices and activities within agencies that were unrelated to the original intervention outcomes.

A few limitations in our study should be noted. First, our narrative themes are based on self-reported sustainment; retrospective assessment years after the fact may be subject to recall bias. We tried to mitigate this limitation by including observed post-intervention wait-time data; however, data from some agencies was lacking. Second, our results may be subject to self-selection bias: some agencies we approached opted out of participating because no current employee remembered the intervention. This could potentially under-report barriers to sustainment; however, we did interview six individuals who revealed they were not at the agency and, thus, captured sustainment from agencies that experienced staff turnover. Interviewing additional agencies who reported no sustainment, or additional agencies who had no staff remaining from the NIATx period, would undoubtedly have revealed additional barriers to sustainment. Our results, therefore, should be interpreted with caution and additional studies should focus on the impact of high staff turnover on QI sustainment. We also attempted to mitigate selection bias by assessing intervention engagement levels between interviewees and non-interviewees and found mixed results. We found no differences between interviewees and non-interviewees based on level of engagement (i.e. session attendance) during the intervention, but did find some differences in post-intervention study engagement (i.e. completing surveys on agency practices) and willingness to participate in an interview. Finally, we were unable to assess the impact of the external environment, including the implementation of the Affordable Care Act, on agency sustainment. Including such information may have produced somewhat different conclusions.

## Conclusions

Some agencies that participated in a quality improvement intervention were able to sustain improvements over a long period of time. Agency capacity—including staff engagement during the intervention, stable staffing afterward, and an investment of human resources to maintain QI practices—were critical to extending intervention effects. Intervention characteristics that aligned with business practices in the agency were also associated with long-term sustainment. Finally, agencies which had the capacity, e.g. a data champion, to embed important intervention characteristics into their organizational QI strategy also showed signs of long-term sustainment.

## Additional file

**Additional file 1.** Current study recruitment diagram from original NIATx200 participation arms.
