# Supplementary material for: Factors influencing the long-term sustainment of quality improvements made in addiction treatment facilities: a qualitative study
Source: Addict Sci Clin Pract. 2017 Nov 1;12:26. doi: 10.1186/s13722-017-0093-x (PMC5664835; doi:10.1186/s13722-017-0093-x)
Supplement: Supplementary file 1 — Additional file 1. Current study recruitment diagram from original NIATx200 participation arms. [file 13722_2017_93_MOESM1_ESM.pdf]

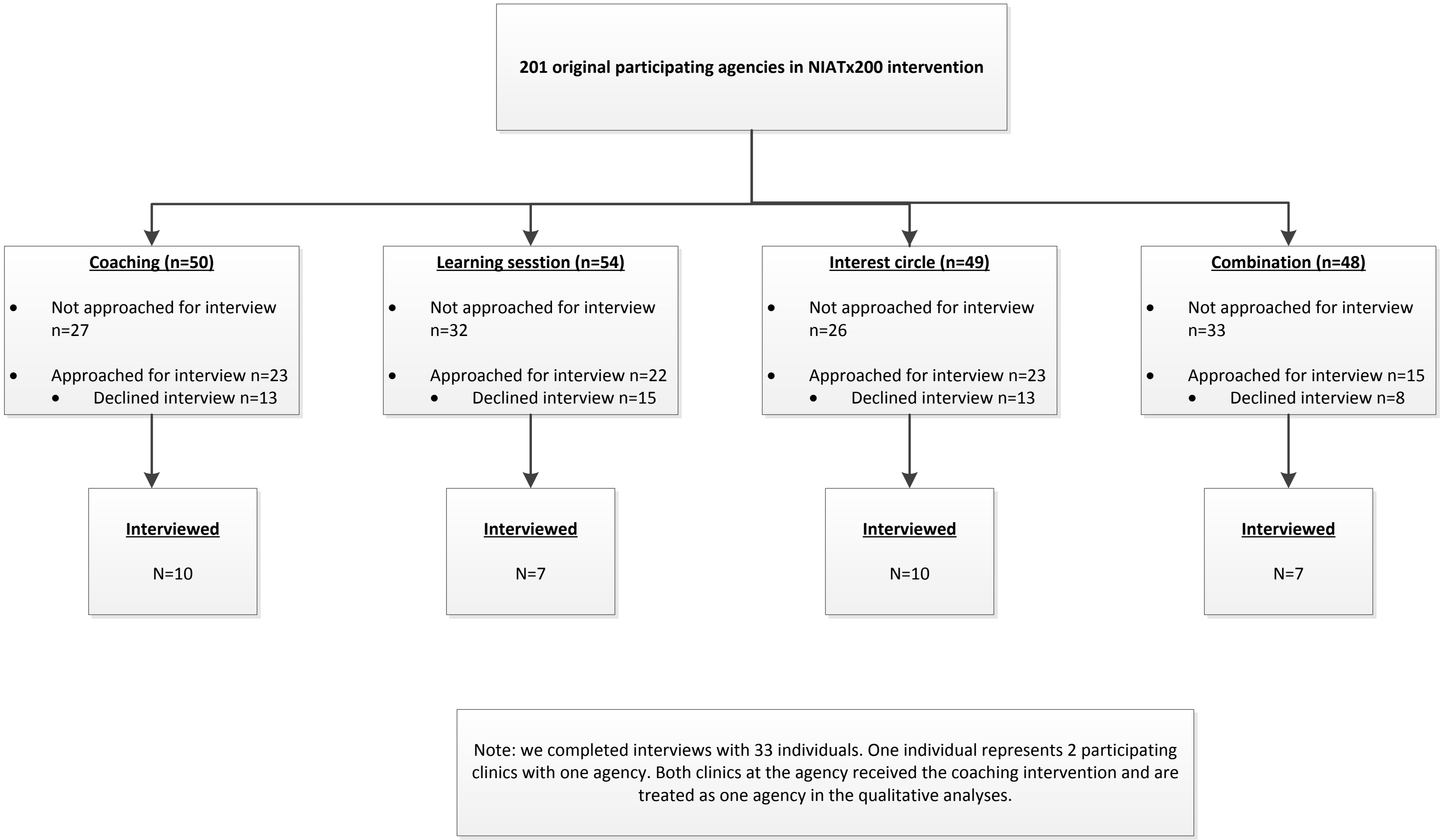
201 original participating agencies in NIATx200 intervention
Coaching (n=50)
Not approached for interview n=27
Approached for interview n=23
Declined interview n=13
Learning sesstion (n=54)
Not approached for interview n=32
Approached for interview n=22
Declined interview n=15
Interest circle (n=49)
Not approached for interview n=26
Approached for interview n=23
Declined interview n=13
Combination (n=48)
Not approached for interview n=33
Approached for interview n=15
Declined interview n=8
Interviewed
N=10
Interviewed
N=7
Interviewed
N=10
Interviewed
N=7
Note: we completed interviews with 33 individuals. One individual represents 2 participating clinics with one agency. Both clinics at the agency received the coaching intervention and are treated as one agency in the qualitative analyses.
